# Retraction: profiling the different needs and expectations of patients for population-based medicine: a case study using segmentation analysis

**DOI:** 10.1186/1472-6963-13-180

**Published:** 2013-05-20

**Authors:** Federico Lega, Alessandro Mengoni

**Affiliations:** 1 Department of Policy Analysis and Public Management, Cergas and Area PMP SDA Bocconi, Bocconi University, Via Rontgen 1, Milan 20136, Italy; 2 ASUR Marche, Via caduti del lavoro 40, Ancona 60100, Italy

## Retraction

This article has been retracted [1] by the authors because they do not have ownership of the data presented, which was originally collected by the Laboratorio Management e Sanità of Scuola Superiore Sant’Anna. In addition, the data presented in this article has previously been used for a publication in the Italian journal “Mercati e Competitività” [2]. The authors apologise to the readers, the publisher and the authors of the Italian publication for any inconvenience caused.

## Pre-publication history

The pre-publication history for this paper can be accessed here:

http://www.biomedcentral.com/1472-6963/13/180/prepub
